# Factors Involved in Morphogenesis in the Muscle–Tendon–Bone Complex

**DOI:** 10.3390/ijms22126365

**Published:** 2021-06-14

**Authors:** Shinichi Abe, Masahito Yamamoto

**Affiliations:** Department of Anatomy, Tokyo Dental College, 2-9-18 Kanda-misakicho, Chiyoda-ku, Tokyo 101-0061, Japan; yamamotomasahito@tdc.ac.jp

**Keywords:** musculotendinous junction, enthesis, Sox9, myostatin

## Abstract

A decline in the body’s motor functions has been linked to decreased muscle mass and function in the oral cavity and throat; however, aging of the junctions of the muscles and bones has also been identified as an associated factor. Basic and clinical studies on the muscles, tendons and bones, each considered independently, have been published. In recent years, however, research has focused on muscle attachment as the muscle–tendon–bone complex from various perspectives, and there is a growing body of knowledge on SRY-box9 (Sox9) and Mohawk(Mkx), which has been identified as a common controlling factor and a key element. Myostatin, a factor that inhibits muscle growth, has been identified as a potential key element in the mechanisms of lifetime structural maintenance of the muscle–tendon–bone complex. Findings in recent studies have also uncovered aspects of the mechanisms of motor organ complex morphostasis in the superaged society of today and will lay the groundwork for treatments to prevent motor function decline in older adults.

## 1. Introduction

Recently, sarcopenia, decreased muscle strength with age, has drawn increasing attention. Sarcopenia causes muscle functional decline, such as decreased grip strength and walking speed, and is proposed as a concept to describe decreased muscle mass in the aged body [1]. The term to describe such an age-related decrease in muscle mass was coined by Rosenberg in 1989, combining the Greek terms sarx (flesh) and penia (loss) [2]. However, the functional decrease with aging is related not only to muscle mass and strength but also to the effects of age-related structural changes in various tissues connected to the muscles. The musculoskeletal system mainly comprises muscles, tendons, ligaments and bones and refers to the system of muscles, tendons, ligaments, bones, joints and related tissues that are used to move the body and maintain its form. The musculotendinous junction, which connects the muscles to tendons, and the enthesis, which connects the tendons to the bones, are essential to physical function. Several studies have treated the “muscle–tendon–bone complex” as a single unit and investigated its mechanisms of morphogenesis and morphostasis; however, many of the details have remained unknown [3].

This article thus summarizes the trends in research on muscle attachment from various perspectives under the premise that understanding the motor organ components of muscle attachment is crucial for uncovering the factors involved in age-related functional decline.

## 2. Elucidating the Process of Musculoskeletal System Morphogenesis

Recent studies from Japan and abroad have shown the special morphogenesis of the musculoskeletal system of the head and neck [4]. They reported a process involving cells derived from the mesoderm of the head and cranial neural crest cells that interact in the mid- and later stages of development. This showed that loss of cranial neural crest cells do not affect the start of normal muscle formation in the head but that the muscle tissues remain small, while the tendons that normally differentiate from the cranial neural crest cells do not. Thus, they do not form the musculotendinous junction during the stage in which desmin aggregates at the muscle bundle end and matures during its formation [5]. That is, the individual tissues of muscle, tendon, ligament and bone that form the musculoskeletal system affect one another to create this functionally important complex. Several studies have provided gradual and partial evidence of this morphogenesis. Here, we introduce several of the subsequent studies and their findings.

The musculotendinous junction at the origin of the superior rectus matures using the vaginae nervi optici as the scaffold. It was found that it is not involved in the ultimate composition of the common tendinous ring [6]. The eyes can move freely in the orbit and change their directions. This is made possible by the mobile end of the extraocular muscles (superior rectus, inferior rectus, lateral rectus, medial rectus, superior oblique, inferior oblique) attaching effectively to the eyeball to control its movement. Tendons at the origin of the extraocular muscles have a secure structure to control the movements. This mechanism is suitable as a model for researching the morphogenesis of the muscle–tendon–bone and resulted in two important findings. First, tendons of the superior rectus that turn the eyeball upward are attached to the vaginae nervi optici and mature the musculotendinous junction structure. Second, it was discovered that the superior rectus muscle transfers its origin to the sphenoid bone in the subsequent stage. Many books have explained that the origin of the extraocular muscles (superior rectus, inferior rectus, lateral rectus and medial rectus) posterior to the eyeball forms the common tendinous ring, but this recent study concluded that the upper rectus is not involved in the formation of the common tendinous ring. That is, the upper rectus has common tendons with the levator muscle of the upper eyelid, suggesting that its functions may operate in conjunction with the function of turning the eye and eyelid upward.

It has been shown that the morphogenesis of the tensor veli palatini and pterygoid hamulus starts early and concurrently in the fetal stage, allowing for the chewing and swallowing movements that start in the fetal stage [7]. Jaw movements begin in mice in the mid-fetal stage for functional and morphological preparation to start suckling smoothly after birth. However, the jawbone during this stage is still immature. The question, “Can muscles attached to the jawbone contract properly even though the jawbone, which it is anchored on, is immature?” is written in the Introduction to that article. In particular, it focused on the developmental process of the bones associated with the tensor veli palatini muscle, which plays an important role. It was shown previously that the palatine aponeurosis/medial plate of the pterygoid process and the tensor veli palatini muscle are formed from the dorsal mesoderm. However, there are no existing reports on the developmental process of the trochleal structure centered on the pterygoid hamulus necessary to tense the veli palatini, which is the most interesting point for understanding the swallowing function. One study revealed that on fetal day 16.5, the tensor veli palatini muscle and palatine aponeurosis form a muscle–tendon junction and that the pterygoid process increases in long and wide diameters with union of the muscle and tendon. In this stage, mice in the fetal stage begin swallowing-like movements, and this suggests that a structure that serves the functions of the veli palatini is created as the function develops even if the bone near the scaphoid fossa, which is the origin, is immature.

A study observed the union of the precursor cell groups of the tendon–skeletal system and muscle anlagen over time in the temporomandibular joint of fetal stage mice and reported the mechanisms of the tissue construction of the motor organs [8]. That paper reported that SRY-box9(Sox9)-positive cells were found in bone and tendon in the early fetal stage but also expressed in the muscle areas of the muscle–tendon junctions. When Sox9 in the Wit-1 region was selectively knocked out, abnormalities were observed in the jawbone and muscle tissues, providing evidence that Sox9 may be involved in all areas of the morphological system forming the muscle–tendon–bone complex.

The micromorphology of the bone at the muscle attachment, which founds the center of motor organs, is known to change with differences in muscle load. However, previous studies have not investigated the effects on muscle function of the morphological changes of soft tissues around the bone or on the morphology of the whole bone to which they are attached. Yamamoto et al. [9] studied the tensor veli palatini muscle in two lines of mice with 98.6% common DNA sequences to observe the effects of differences in muscle morphology, in other words, differences in the functional load from the muscles on bone morphogenesis and maintenance. At 10 days after birth, differences in the angle of the tensor veli palatini muscle attached to the sphenoid bone and the morphology of the adjacent pterygoid process were observed. Yamamoto et al. thus showed that differences in muscle tissue and bone morphology were observed at the same time in the developmental stage and that morphogenesis occurred through their interactions. Moreover, they pointed out the importance of maintaining the stability of the muscle and other soft tissues attached to bone tissue to maintain the structure of the tissue complex formed by the bones and attached muscles.

## 3. Trends in Research on Musculoskeletal System Morphogenesis

The musculoskeletal system is a tissue essential for moving the body and stabilizing physical function. This musculoskeletal system mainly comprises muscles, tendons, ligaments and bones, each with their distributions of nerves and blood vessels [10]. One of the musculoskeletal systems in the head and neck region is the temporomandibular joint, which functions with the attachment of the lateral pterygoid muscle to the lower jaw and articular disc. That is, various tissues, such as bone, muscle, tendon and articular disc, work in a coordinated manner to produce jaw movement [11]. Although previous studies have made morphological observations focusing on the lower jaw and some surrounding soft tissues as they relate to the development of the temporomandibular joint, none have detailed the interactions between the developmental stages of the individual tissues. Tendons and ligaments are rich in type I collagen and are organized in a regular pattern to form fibrous connective tissues. Load is always exerted on entheses in physical movement, and they are more prone to degenerative changes related to externally acquired injury or age [12]. Entheses have poor vasculature; thus, they are difficult to regenerate into the original form after injury. In other words, maintenance or regeneration of entheses is important during the care of older adults; however, it is difficult based on the current knowledge to stimulate the regeneration of damaged entheses. Thus, a better embryological understanding of the enthesis is warranted to improve regenerative medicine for treating the enthesis.

Recently, Subramanian and Schilling reported new roles of the extracellular matrix in the process of maturation of tendons and bones in muscle attachment [13]. That is, they found evidence explaining part of the process of interactions between tissues of different origins to differentiate and mature into a tissue complex in the developmental stages of the musculoskeletal system.

### 3.1. Development of the Musculotendinous Junction

In the musculotendinous junction, the contractility of muscle tissues is conducted by collagen fibers of the tendons so the two are in a functionally close relationship, and the state of the junction of the microstructures of the muscle and tendon has been reported [14]. Abe et al. reported that the intermediate filaments and desmin aggregate to form the morphology at the tendon end of muscles during the developmental process of the musculotendinous junction [5]. The period for desmin aggregation at the muscle end overlaps with when the mesoderm-derived myocyte precursor groups differentiate into individual muscles. This was described by Yamamoto et al., who reported that individual muscles mature after the position of the musculotendinous junction is determined by the precursor cells of the myocytes [15]. Their study found that tendons that differentiated from cranial neural crest cells play an important role in the formation of the musculotendinous junction at sites that are in contact with muscle; in other words, that some of the collagen fibers in the tendinous tissues are inserted into the muscle over time throughout the course of development.

### 3.2. Development of the Enthesis

Entheses are classified into three types: (1) periosteal muscle insertions, (2) fibrous insertions, and (3) fibrocartilage insertions [12].

(1) “Periosteal muscle insertions” means the following: Myofiber bundles are attached directly to the periosteum without transition to tendons. (2) “Fibrous insertions” means the following: Myofiber bundles transition to tendons, and the tendons are directly bound to the periosteum via fibrous tissues. (3) “Fibrocartilage insertions” means the following: Myofiber bundles transition to tendons, and the tendons are directly bound to the periosteum via a fibrocartilage layer.

Collagen fibers in the tendon and ligament tissues in the enthesis are aligned in a regular organization in the direction of the tension from the muscle fiber bundles. The individual fiber bundles are surrounded by endotenons, and the tendons are surrounded by epitenons. Vascular channels and nerves are distributed in both the endotenon and epitenon, playing a role in maintaining the constancy of the tissues and repairing injuries. The main cellular components of the endotenon and epitenon are tenocytes ordered along the collagen fibers. Tenocytes are reported to express tenomodulin (Tnmd), a type II transmembrane glycoprotein [16].

**Figure 1:**
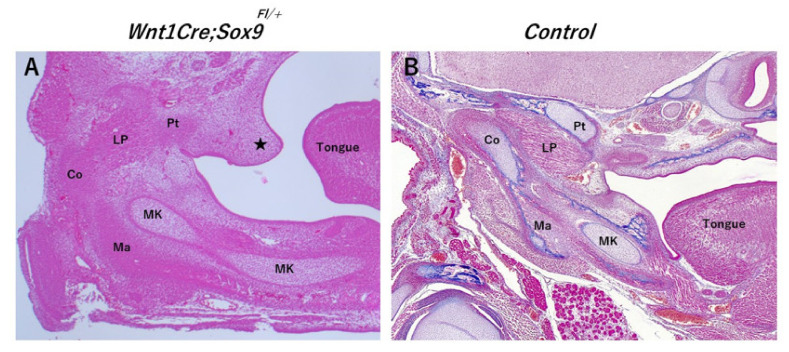
Histological view of WntCre;Sox9fl/+ mice. (**A**) WntCre;Sox9fl/+ mice, (**B**) control mice (C57BL/6J). Deficiency of Sox9 in neural crest-derived cells (Wnt1-positive) results in hypoplasia of the condylar head (Co), mandible (Ma) and lateral pterygoid (LP) in the temporomandibular joint area (**A**). Deficiency of Sox9 in neural crest-derived cells (Wnt1-positive) also results in poor fusion of the palate ((**A**); black star). Co: condylar head, LP: lateral pterygoid, Ma: mandible, MK: Meckel’s cartilage, Pt: pterygoid bone.

Recent studies have focused on the developmental process of fibrocartilage inserts in the enthesis. Among these, fibrocartilage insertions are reported to be formed in precursor cell groups that coexpress Sox9 and a multipotent cell population coexpressing a basic-helix–loop–helix transcription factor scleraxis (Scx) [17]. Furthermore, Scx-deficient ScxCre/Cre KI mice were observed to have hypoplasia of fibrocartilage insertions in the enthesis [18]. According to this report, Sox9 expression was remarkably decreased in fibrocartilage insertions in the enthesis. Therefore, Sox9 and Scx are key factors of enthesis formation (Figure 1). 

TGF-β is a growth factor essential for chondrocyte differentiation. It has also been reported recently that TGF-β effectively promotes tendon and ligament formation through interactions with Sox9, a transcription factor of chondrogenesis [19]. Moreover, Scx expressed via the BMP4 signal is known to be involved in the formation of fibrocartilage insertions in the enthesis [20]. These reports have made it clear that Sox9 and Scx-positive precursor cell groups form fibrocartilage insertions under the control of TGFβ and BMP signals.

## 4. Toward Elucidating the Mechanism of Structural Maintenance of the Muscle–Tendon–Bone Complex

Decreased function of joints such as the temporomandibular joint in older adults is often related to age-related changes in muscle tissues, as well as to structural breakdown of the junction of the muscle bundle ends attached to the tendon and bone. In other words, maintaining the structure of the tissue complexes that form motor organs is the key to preventing functional decline in older adults. Moreover, the intramuscular tendons of the muscle bundle ends formed in the developmental stage constitute the key to the muscle–tendon–bone complex, and it has been reported that Mohawk (Mkx) and myostatin may play important roles in this stage [21,22]. Specifically, this refers to the scenario where: (1) Mkx promotes tendon precursor cells to move freely inside muscles at all times to differentiate into tendon cells, and (2) myostatin stimulates a phenotypic transformation of the myocytes at the end of muscle bundles into tendon cells, thereby strengthening the junction at the muscle attachment. Studies on these topics may promote the development of approaches to prevent motor functional decline in older adults.

### 4.1. Tendon Development and Molecular Mechanisms: Involvement of Mkx

When a tendon is damaged, the mechanism for promoting its repair has not been fully elucidated. Ito et al. used CRISPR/Cas to generate Mkx^−/−^ rats and showed early ectopic ossification in the Achilles tendon as well as systemic tendon hypoplasia. Among them, it was confirmed that Mkx deficiency promotes chondrogenesis and bone formation, and conversely, it was clarified that overexpression of Mkx suppresses chondrogenesis and bone formation [21]. In other words, Mkx has two roles: acceleration and suppression of tendon differentiation. This suggests that Mkx may be deeply involved in the morphogenesis and maintenance of the “muscle–tendon–bone complex”.

### 4.2. Characterization of Lifetime Musculotendinous Junction Structure Maintenance by Myostatin

**Figure 2:**
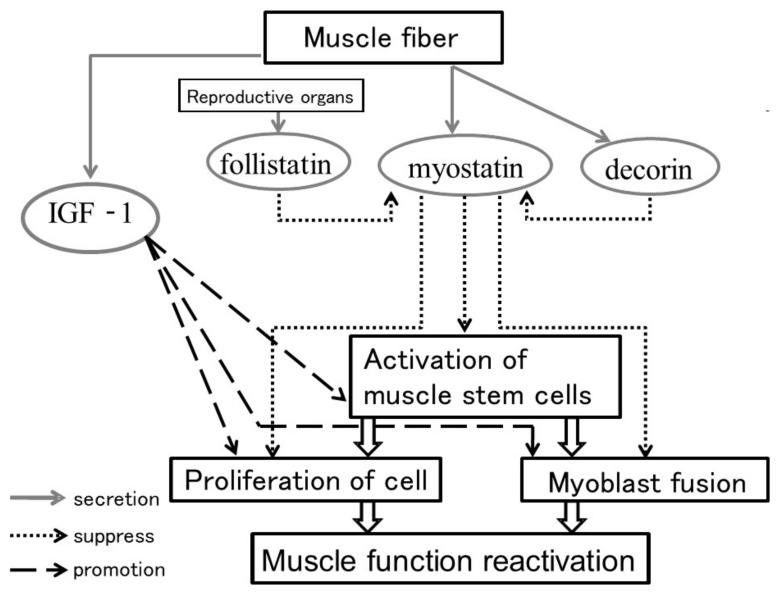
Factors associated with muscle development and hypertrophy. Growth factors such as IGF-1 promote the proliferation and fusion of myoblasts and promote the activity and self-renewal of satellite cells, which are muscle stem cells, thereby promoting muscle development and muscle hypertrophy [23]. On the other hand, it has been reported that myostatin secreted from muscle cells suppresses the proliferation and fusion of myoblasts and suppresses the activity and self-renewal of satellite cells, thereby suppressing muscle development and muscle hypertrophy [25,26,27]. Furthermore, the function of follistatin/decorin [28,29], which regulates the function of myostatin, has also been reported. Follistatin inhibits the action of myostatin by binding to myostatin [28]. Decorin is a protein produced by muscle cells at the same time as myostatin, and it has been reported that it regulates the activity of myostatin by binding to myostatin [29].

The molecular mechanism of sarcopenia can be explained by findings from general animal experiments showing the atrophy and loss of type IIb fibers, which are isoforms of type II fast muscles (white muscles). Proteins that serve various roles in muscle tissues are constantly broken down and newly synthesized. Furthermore, the muscle mass is maintained at a constant level through a balance between self-renewal and self-regeneration of satellite cells or muscle resident stem cells [23,24]. Myostatin is a factor that has the opposite effect of factors involved in muscle development, maturation, and hypertrophy [25,26,27], such as IGF-1 [23]. That is, it inhibits muscle hypertrophy while interacting with follistatin/decorin [28,29] (Figure 2). In recent years, myostatin, which functions as an inhibitor of such muscle activity, was shown to play a potentially important role in maintaining the structure of the musculotendinous junction in an experiment using mice [22]. That is, although the muscle weight temporarily increases by inhibiting myostatin expression, it weakens the tendon at the muscle attachment, preventing muscle strength from increasing in the long term or in any lasting way. This finding will likely become an important key to understanding how to maintain the musculotendinous junction structure in older adults. One experiment with rats [30] showed that even if myostatin is reduced, there is no abnormality in the tendon at the “muscle–tendon junction”; however, the authors did not perform a thorough histological analysis of the junction. This needs to be carried out in the future.

### 4.3. Role of Sox9 in the “Muscle–Tendon–Bone Complex” in the Elderly

In previous research, Scx and Sox9 have been shown to contribute to the establishment of tendon attachment sites (entheses) during embryonic development (using mice). Ideo et al. damaged the muscle–tendon–bone complex (entheses of the supraspinatus tendon) and tested for Scx+/Sox9+ cells in injured young and 20-week-old mice. Scx+/Sox9+ cells were predominantly expressed in the injured part in young mice compared to 20-week-old mice, and the healing process was the same as in the embryonic period, but the healing process was more complete in young mice. This ability showed superiority [31]. This suggests that in the muscle–tendon–bone complex in the elderly, the expression of Scx+/Sox9+ cells for remodeling is weak, and its maintenance ability of the structure may consequently be weakened.

### 4.4. Connection at the Muscle–Tendon Junction

Tendons that extend into the muscles are called intramuscular tendons. In contrast, tendons such as the Achilles tendon are classified as extramuscular tendons. Although the collagen structures of intra- and extramuscular tendons are similar, they are different in their healing course after an injury [32]. Furthermore, intramuscular tendon injury prolongs the time to recover after an acute hamstring injury [33]. Because the tendon tissue at the muscle attachment is continuous with the connective tissue within the muscle, damage to the muscle attachment should be considered to be damage to the muscle–tendon–bone complex, unlike damage to muscle cells alone.

## 5. Muscle Tonus Load on the Enthesis and Aging of the Muscle–Tendon Junction

Contraction of the skeletal muscles generates tonus that is then transmitted to the tendons. Because the skeletal muscles are attached to bone on both sides, torque is generated in the joint contained between the two edges of the bone. The contraction characteristics of individual muscle fibers are determined by each muscle fiber type and size. Muscle fibers have a diameter of 10 to 100 μm, and their length ranges from several mm to 10 cm. These muscle fibers contain myofibrils that have diameters of 0.5 to 2 μm. Myofibrils are made up of thick filaments, mainly comprising myosin, and fine filaments, mainly comprising actin. These slide against each other to produce skeletal muscle contraction [34]. To date, only muscle tissue structure and muscle fiber types have been discussed when considering muscle function. However, the locomotorium forms and functions as a muscle–tendon–bone complex.

### 5.1. Muscle Fiber Paths and Muscle Tonus

Skeletal muscle contractive force is known to differ from contraction speed since longer muscle fibers with a smaller cross-sectional area of the muscle are associated with a higher contraction speed. However, a shorter muscle fiber length and a larger cross-sectional area of the muscle are advantageous for producing maximum muscle strength [35]. Interestingly, this report suggested that the human gastrocnemius muscle has many oblique fibers and a large muscle fiber cross-sectional area, enabling strong muscle strength to be produced throughout the muscle tissue overall.

### 5.2. Muscle Tonus Stimulation and Changes to Muscle Contraction Protein in the Enthesis

General muscle strength training results in increased muscle mass. This is most markedly observed in the enthesis or distal point of each muscle [36]. This phenomenon is thought to be caused by stimulation from muscle tonus mediating the sarcolemma and basal lamina located between the muscle and tendon.

### 5.3. Functional Differences Related to Muscle Type

Changes in muscle tonus associated with muscle fiber length are described above. Considering this relationship, tonus can be displayed over a wider range for muscles such as smooth muscles that have less clear-cut structures than skeletal muscles and are not striated muscles. In the stomach, for example, the entry of food causes the stomach to expand, and it then returns to its original size when empty. The maximum speed of skeletal muscle contraction without load differs depending on the muscle. This speed is determined by the properties of myosin protein, which oversees muscle contraction. This phenomenon also affects the distribution of blood flow [37]. Skeletal muscles can alter their overall role by altering the composition ratio of fast twitch fibers, which have a fast contraction speed; red muscle fibers, which are slower, and intermediate muscle fibers [38,39,40,41,42,43]. 

As smooth muscles break down ATP and have a very slow cycle for producing force, their contraction speed is slower when no load is applied. As a slow contraction speed means that more time is required to produce the force, this force can be produced with less energy. Thus, muscles with functions that do not require much speed have a different structure from muscles that mainly function to move bones.

## 6. Mkx, Myostatin and Sox9 Are Expressed during the Musculoskeletal Development

**Figure 3:**
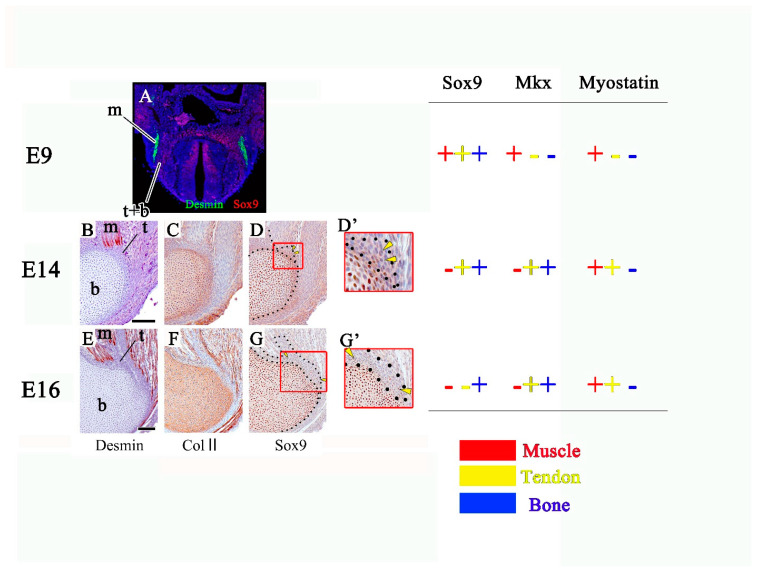
Mkx, myostatin and Sox9 are expressed during the musculoskeletal development. (**A**) Transverse section of embryo at E9. (**B**–**G**) Sagittal sections of attachment site of the triceps brachii muscle at E14 and 16. (**D’**,**G’**) is the high magnification view of (**D**,**G**). m: future muscle, t: future tendon, b: future bone. (Scale bar:100 μm).

Mkx, myostatin and Sox9 are known to be expressed in components of the musculoskeletal system (Figure 3). Mkx regulates early myogenesis in vertebrates and invertebrates [44,45]. Then, it is expressed throughout the anlage of the tendon and cartilage positioned between the myotome [43]. Mkx mutant mice and rats show that Mkx accelerates tendon differentiation and prevents chondrogenic/osteogenic differentiation [21,30]. On the other hand, myostatin affects the balance between proliferation and differentiation of embryonic muscle progenitors [46]. Myostatin is a potent regulator of tendon cell proliferation in vitro [47,48,49], but it is unclear whether it regulates tendon cell differentiation and proliferation in vivo. Moreover, myostatin inhibits chondrogenesis and chondrocyte proliferation by suppressing Sox9 [50]. Sox9 is expressed in progenitor cells of all components of the musculoskeletal system and then detected in the tendon and bone. Finally, bones only show Sox9 expression [8]. 

## 7. Conclusions

The attachments of muscles described in this article are specialized areas that connect skeletal muscle and tendinous tissues. This junction transmits the contractility produced by skeletal muscle tissues to collagen fibers of the tendons, and they are in close functional interactions with each other. That is, on the muscle side, the sarcolemma, which is a cell membrane that surrounds skeletal muscle fibers, presents an interdigitation to increase the attachment surface, and on the tendon side, the collagen fibers produced by the tenocytes that exist in the tendinous cells are anchored perpendicularly to the sarcolemma. This interdigitation helps resist muscular contractility and transmits it to the tendons. This concludes this review of developmental research on muscle, tendon and bone considered as one unit, which explains the facts that were already proven on the microstructural level. Sox9, Scx, Mkx, etc. have been identified as important factors in the formation process of this muscle–tendon–bone complex, and these factors are also key factors in the repair processes after injury of the complex. The muscle–tendon–bone complex is remodeled like muscle and bone throughout life. Maintaining the function of this locomotorium in the elderly may be extremely important, and it will be an important consideration in the management of various disorders of the elderly, such as sarcopenia.

## Data Availability

Not applicable.
